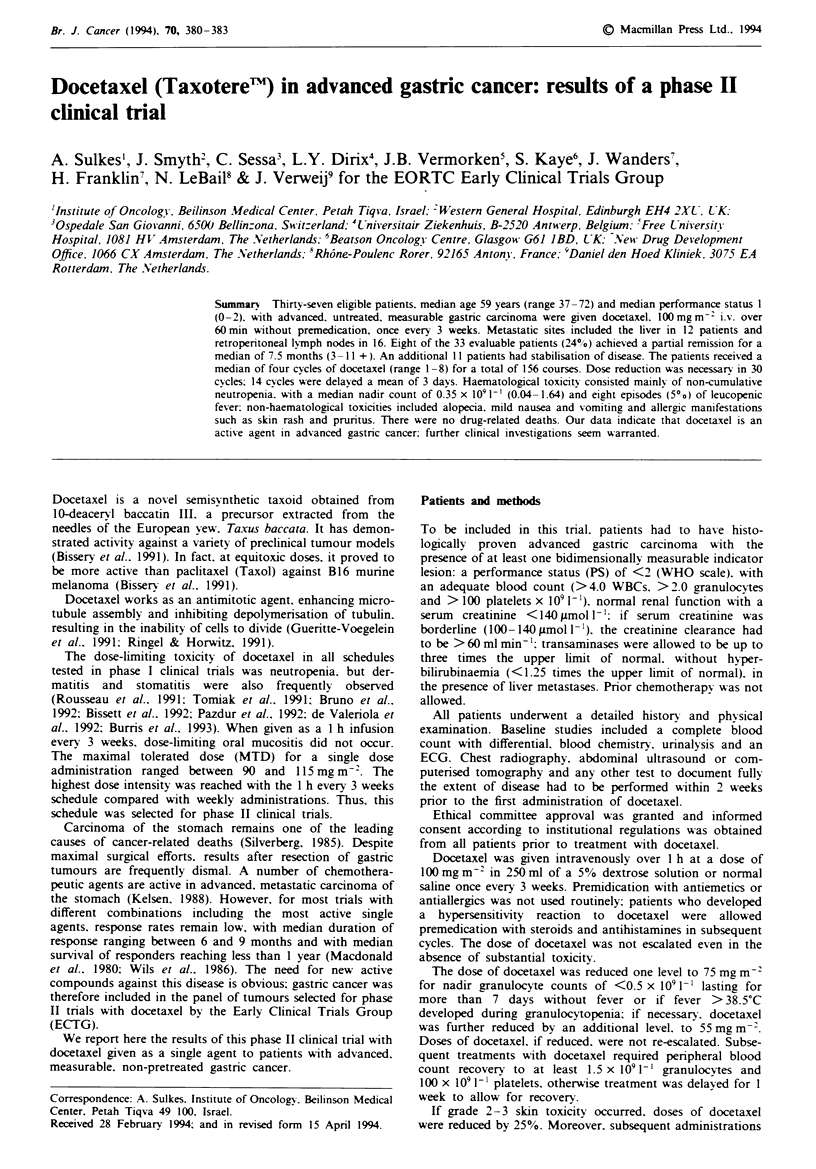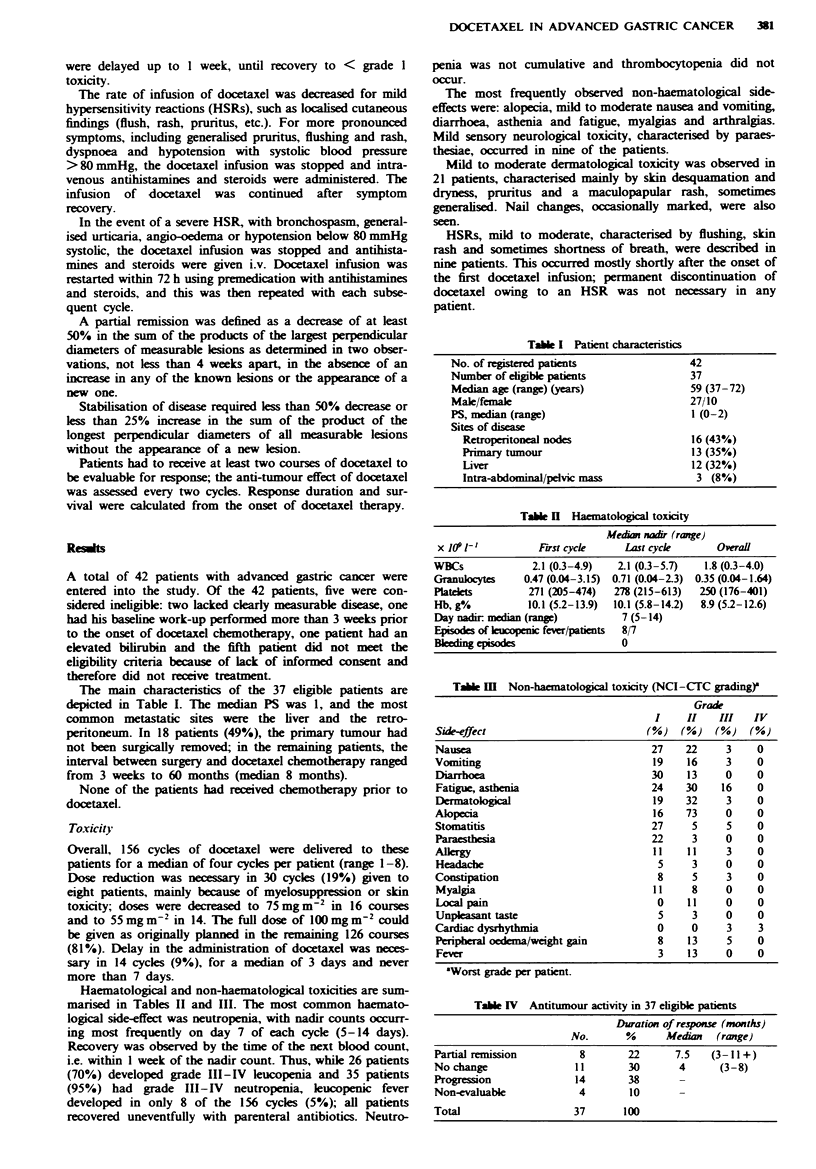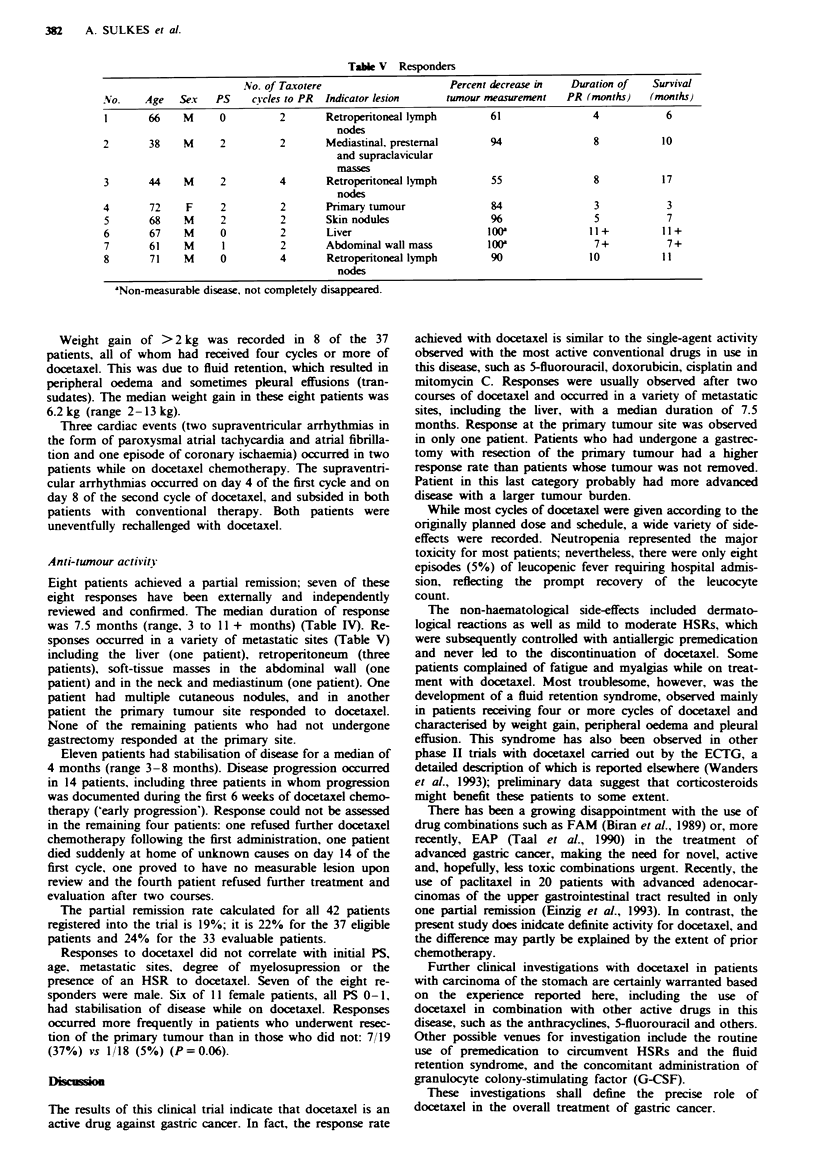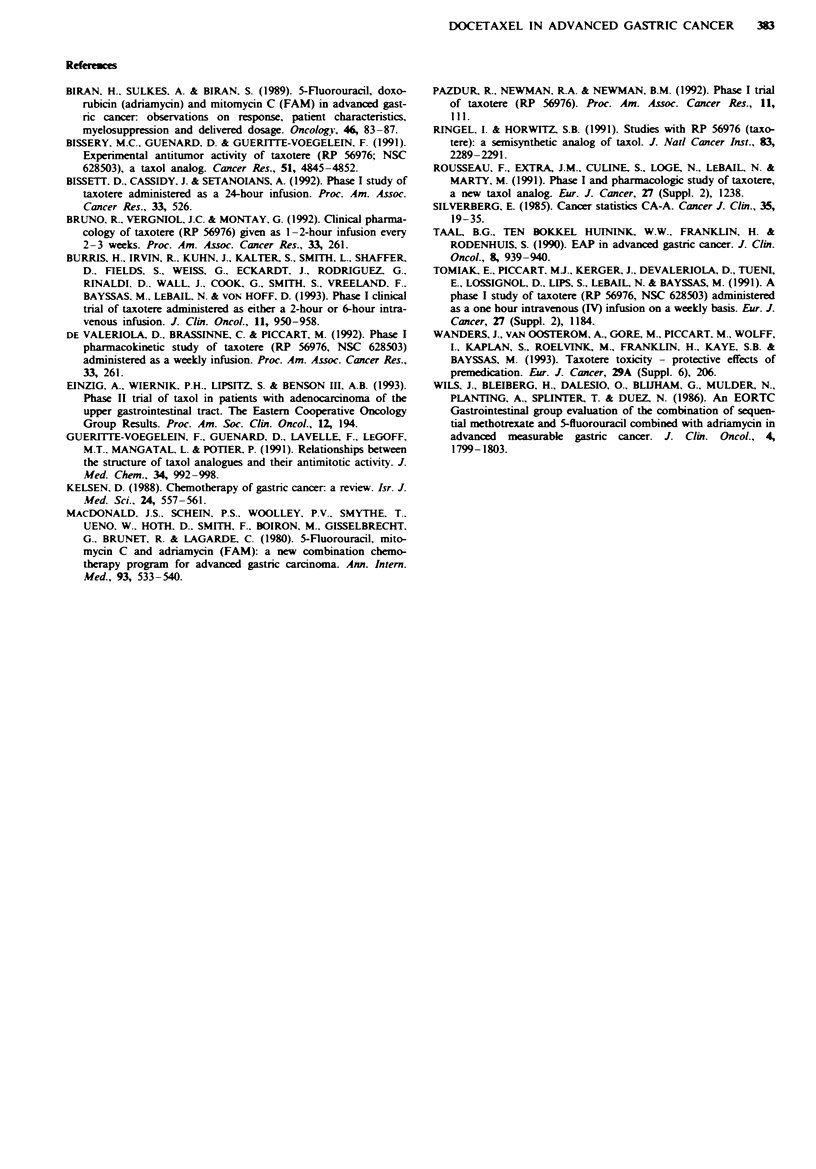# Docetaxel (Taxotere) in advanced gastric cancer: results of a phase II clinical trial. EORTC Early Clinical Trials Group.

**DOI:** 10.1038/bjc.1994.310

**Published:** 1994-08

**Authors:** A. Sulkes, J. Smyth, C. Sessa, L. Y. Dirix, J. B. Vermorken, S. Kaye, J. Wanders, H. Franklin, N. LeBail, J. Verweij

**Affiliations:** Institute of Oncology, Beilinson Medical Center, Petah Tiqva, Israel.

## Abstract

Thirty-seven eligible patients, median age 59 years (range 37-72) and median performance status 1 (0-2), with advanced, untreated, measurable gastric carcinoma were given docetaxel, 100 mg m-2 i.v. over 60 min without premedication, once every 3 weeks. Metastatic sites included the liver in 12 patients and retroperitoneal lymph nodes in 16. Eight of the 33 evaluable patients (24%) achieved a partial remission for a median of 7.5 months (3-11+). An additional 11 patients had stabilisation of disease. The patients received a median of four cycles of docetaxel (range 1-8) for a total of 156 courses. Dose reduction was necessary in 30 cycles; 14 cycles were delayed a mean of 3 days. Haematological toxicity consisted mainly of non-cumulative neutropenia, with a median nadir count of 0.35 x 10(9) l-1 (0.04-1.64) and eight episodes (5%) of leucopenic fever; non-haematological toxicities included alopecia, mild nausea and vomiting and allergic manifestations such as skin rash and pruritus. There were no drug-related deaths. Our data indicate that docetaxel is an active agent in advanced gastric cancer; further clinical investigations seem warranted.


© Macmillan Press Ltd., 1994

### Docetaxel (Taxotere™) in advanced gastric cancer: results of a phase II clinical trial

A. Sulkes[1], J. Smyth[2], C. Sessa[3], L.Y. Dirix[4], J.B. Vermorken[5], S. Kaye[6], J. Wanders[7], H. Franklin[7], N. LeBail[8] & J. Verweij[9] for the EORTC Early Clinical Trials Group

[1]*Institute of Oncology, Beilinson Medical Center, Petah Tiqva, Israel;* [2]*Western General Hospital, Edinburgh EH4 2XU, UK;* [3]*Ospedale San Giovanni, 6500 Bellinzona, Switzerland;* [4]*Universitair Ziekenhuis, B-2520 Antwerp, Belgium;* [5]*Free University Hospital, 1081 HV Amsterdam, The Netherlands;* [6]*Beatson Oncology Centre, Glasgow G61 1BD, UK;* [7]*New Drug Development Office, 1066 CX Amsterdam, The Netherlands;* [8]*Rhône-Poulenc Rorer, 92165 Antony, France;* [9]*Daniel den Hoed Kliniek, 3075 EA Rotterdam, The Netherlands.*

**Summary** Thirty-seven eligible patients, median age 59 years (range 37–72) and median performance status 1 (0–2), with advanced, untreated, measurable gastric carcinoma were given docetaxel, 100 mg m$^{-2}$ i.v. over 60 min without premedication, once every 3 weeks. Metastatic sites included the liver in 12 patients and retroperitoneal lymph nodes in 16. Eight of the 33 evaluable patients (24%) achieved a partial remission for a median of 7.5 months (3–11 +). An additional 11 patients had stabilisation of disease. The patients received a median of four cycles of docetaxel (range 1–8) for a total of 156 courses. Dose reduction was necessary in 30 cycles; 14 cycles were delayed a mean of 3 days. Haematological toxicity consisted mainly of non-cumulative neutropenia, with a median nadir count of $0.35 \times 10^9$ l$^{-1}$ (0.04–1.64) and eight episodes (5%) of leucopenic fever; non-haematological toxicities included alopecia, mild nausea and vomiting and allergic manifestations such as skin rash and pruritus. There were no drug-related deaths. Our data indicate that docetaxel is an active agent in advanced gastric cancer; further clinical investigations seem warranted.

Docetaxel is a novel semisynthetic taxoid obtained from 10-deaceryl baccatin III, a precursor extracted from the needles of the European yew, *Taxus baccata*. It has demonstrated activity against a variety of preclinical tumour models (Bissery *et al.*, 1991). In fact, at equitoxic doses, it proved to be more active than paclitaxel (Taxol) against B16 murine melanoma (Bissery *et al.*, 1991).

Docetaxel works as an antimitotic agent, enhancing microtubule assembly and inhibiting depolymerisation of tubulin, resulting in the inability of cells to divide (Gueritte-Voegelein *et al.*, 1991; Ringel & Horwitz, 1991).

The dose-limiting toxicity of docetaxel in all schedules tested in phase I clinical trials was neutropenia, but dermatitis and stomatitis were also frequently observed (Rousseau *et al.*, 1991; Tomiak *et al.*, 1991; Bruno *et al.*, 1992; Bissett *et al.*, 1992; Pazdur *et al.*, 1992; de Valeriola *et al.*, 1992; Burris *et al.*, 1993). When given as a 1 h infusion every 3 weeks, dose-limiting oral mucositis did not occur. The maximal tolerated dose (MTD) for a single dose administration ranged between 90 and 115 mg m$^{-2}$. The highest dose intensity was reached with the 1 h every 3 weeks schedule compared with weekly administrations. Thus, this schedule was selected for phase II clinical trials.

Carcinoma of the stomach remains one of the leading causes of cancer-related deaths (Silverberg, 1985). Despite maximal surgical efforts, results after resection of gastric tumours are frequently dismal. A number of chemotherapeutic agents are active in advanced, metastatic carcinoma of the stomach (Kelsen, 1988). However, for most trials with different combinations including the most active single agents, response rates remain low, with median duration of response ranging between 6 and 9 months and with median survival of responders reaching less than 1 year (Macdonald *et al.*, 1980; Wils *et al.*, 1986). The need for new active compounds against this disease is obvious; gastric cancer was therefore included in the panel of tumours selected for phase II trials with docetaxel by the Early Clinical Trials Group (ECTG).

We report here the results of this phase II clinical trial with docetaxel given as a single agent to patients with advanced, measurable, non-pretreated gastric cancer.

Correspondence: A. Sulkes, Institute of Oncology, Beilinson Medical Center, Petah Tiqva 49 100, Israel.

Received 28 February 1994; and in revised form 15 April 1994.

### Patients and methods

To be included in this trial, patients had to have histologically proven advanced gastric carcinoma with the presence of at least one bidimensionally measurable indicator lesion; a performance status (PS) of $<2$ (WHO scale), with an adequate blood count ($>4.0$ WBCs, $>2.0$ granulocytes and $>100$ platelets $\times 10^9$ l$^{-1}$), normal renal function with a serum creatinine $<140$ µmol l$^{-1}$; if serum creatinine was borderline (100–140 µmol l$^{-1}$), the creatinine clearance had to be $>60$ ml min$^{-1}$; transaminases were allowed to be up to three times the upper limit of normal, without hyperbilirubinaemia ($<1.25$ times the upper limit of normal), in the presence of liver metastases. Prior chemotherapy was not allowed.

All patients underwent a detailed history and physical examination. Baseline studies included a complete blood count with differential, blood chemistry, urinalysis and an ECG. Chest radiography, abdominal ultrasound or computerised tomography and any other test to document fully the extent of disease had to be performed within 2 weeks prior to the first administration of docetaxel.

Ethical committee approval was granted and informed consent according to institutional regulations was obtained from all patients prior to treatment with docetaxel.

Docetaxel was given intravenously over 1 h at a dose of 100 mg m$^{-2}$ in 250 ml of a 5% dextrose solution or normal saline once every 3 weeks. Premidication with antiemetics or antiallergics was not used routinely; patients who developed a hypersensitivity reaction to docetaxel were allowed premedication with steroids and antihistamines in subsequent cycles. The dose of docetaxel was not escalated even in the absence of substantial toxicity.

The dose of docetaxel was reduced one level to 75 mg m$^{-2}$ for nadir granulocyte counts of $<0.5 \times 10^9$ l$^{-1}$ lasting for more than 7 days without fever or if fever $>38.5$°C developed during granulocytopenia; if necessary, docetaxel was further reduced by an additional level, to 55 mg m$^{-2}$. Doses of docetaxel, if reduced, were not re-escalated. Subsequent treatments with docetaxel required peripheral blood count recovery to at least $1.5 \times 10^9$ l$^{-1}$ granulocytes and $100 \times 10^9$ l$^{-1}$ platelets, otherwise treatment was delayed for 1 week to allow for recovery.

If grade 2–3 skin toxicity occurred, doses of docetaxel were reduced by 25%. Moreover, subsequent administrations were delayed up to 1 week, until recovery to < grade 1 toxicity.

The rate of infusion of docetaxel was decreased for mild hypersensitivity reactions (HSRs), such as localised cutaneous findings (flush, rash, pruritus, etc.). For more pronounced symptoms, including generalised pruritus, flushing and rash, dyspnoea and hypotension with systolic blood pressure > 80 mmHg, the docetaxel infusion was stopped and intravenous antihistamines and steroids were administered. The infusion of docetaxel was continued after symptom recovery.

In the event of a severe HSR, with bronchospasm, generalised urticaria, angio-oedema or hypotension below 80 mmHg systolic, the docetaxel infusion was stopped and antihistamines and steroids were given i.v. Docetaxel infusion was restarted within 72 h using premedication with antihistamines and steroids, and this was then repeated with each subsequent cycle.

A partial remission was defined as a decrease of at least 50% in the sum of the products of the largest perpendicular diameters of measurable lesions as determined in two observations, not less than 4 weeks apart, in the absence of an increase in any of the known lesions or the appearance of a new one.

Stabilisation of disease required less than 50% decrease or less than 25% increase in the sum of the product of the longest perpendicular diameters of all measurable lesions without the appearance of a new lesion.

Patients had to receive at least two courses of docetaxel to be evaluable for response; the anti-tumour effect of docetaxel was assessed every two cycles. Response duration and survival were calculated from the onset of docetaxel therapy.

### Results

A total of 42 patients with advanced gastric cancer were entered into the study. Of the 42 patients, five were considered ineligible: two lacked clearly measurable disease, one had his baseline work-up performed more than 3 weeks prior to the onset of docetaxel chemotherapy, one patient had an elevated bilirubin and the fifth patient did not meet the eligibility criteria because of lack of informed consent and therefore did not receive treatment.

The main characteristics of the 37 eligible patients are depicted in Table I. The median PS was 1, and the most common metastatic sites were the liver and the retroperitoneum. In 18 patients (49%), the primary tumour had not been surgically removed; in the remaining patients, the interval between surgery and docetaxel chemotherapy ranged from 3 weeks to 60 months (median 8 months).

None of the patients had received chemotherapy prior to docetaxel.

### *Toxicity*

Overall, 156 cycles of docetaxel were delivered to these patients for a median of four cycles per patient (range 1–8). Dose reduction was necessary in 30 cycles (19%) given to eight patients, mainly because of myelosuppression or skin toxicity; doses were decreased to 75 mg m$^{-2}$ in 16 courses and to 55 mg m$^{-2}$ in 14. The full dose of 100 mg m$^{-2}$ could be given as originally planned in the remaining 126 courses (81%). Delay in the administration of docetaxel was necessary in 14 cycles (9%), for a median of 3 days and never more than 7 days.

Haematological and non-haematological toxicities are summarised in Tables II and III. The most common haematological side-effect was neutropenia, with nadir counts occurring most frequently on day 7 of each cycle (5–14 days). Recovery was observed by the time of the next blood count, i.e. within 1 week of the nadir count. Thus, while 26 patients (70%) developed grade III–IV leucopenia and 35 patients (95%) had grade III–IV neutropenia, leucopenic fever developed in only 8 of the 156 cycles (5%); all patients recovered uneventfully with parenteral antibiotics. Neutropenia was not cumulative and thrombocytopenia did not occur.

The most frequently observed non-haematological side-effects were: alopecia, mild to moderate nausea and vomiting, diarrhoea, asthenia and fatigue, myalgias and arthralgias. Mild sensory neurological toxicity, characterised by paraesthesiae, occurred in nine of the patients.

Mild to moderate dermatological toxicity was observed in 21 patients, characterised mainly by skin desquamation and dryness, pruritus and a maculopapular rash, sometimes generalised. Nail changes, occasionally marked, were also seen.

HSRs, mild to moderate, characterised by flushing, skin rash and sometimes shortness of breath, were described in nine patients. This occurred mostly shortly after the onset of the first docetaxel infusion; permanent discontinuation of docetaxel owing to an HSR was not necessary in any patient.

**Table I** Patient characteristics

| | |
|---|---|
| No. of registered patients | 42 |
| Number of eligible patients | 37 |
| Median age (range) (years) | 59 (37–72) |
| Male/female | 27/10 |
| PS, median (range) | 1 (0–2) |
| Sites of disease | |
| Retroperitoneal nodes | 16 (43%) |
| Primary tumour | 13 (35%) |
| Liver | 12 (32%) |
| Intra-abdominal/pelvic mass | 3 (8%) |

**Table II** Haematological toxicity

| | Median nadir (range) | | |
|---|---|---|---|
| $\times 10^9 l^{-1}$ | First cycle | Last cycle | Overall |
| WBCs | 2.1 (0.3–4.9) | 2.1 (0.3–5.7) | 1.8 (0.3–4.0) |
| Granulocytes | 0.47 (0.04–3.15) | 0.71 (0.04–2.3) | 0.35 (0.04–1.64) |
| Platelets | 271 (205–474) | 278 (215–613) | 250 (176–401) |
| Hb, g% | 10.1 (5.2–13.9) | 10.1 (5.8–14.2) | 8.9 (5.2–12.6) |
| Day nadir: median (range) | | 7 (5–14) | |
| Episodes of leucopenic fever/patients | | 8/7 | |
| Bleeding episodes | | 0 | |

**Table III** Non-haematological toxicity (NCI–CTC grading)[a]

| Side-effect | Grade I (%) | II (%) | III (%) | IV (%) |
|---|---|---|---|---|
| Nausea | 27 | 22 | 3 | 0 |
| Vomiting | 19 | 16 | 3 | 0 |
| Diarrhoea | 30 | 13 | 0 | 0 |
| Fatigue, asthenia | 24 | 30 | 16 | 0 |
| Dermatological | 19 | 32 | 3 | 0 |
| Alopecia | 16 | 73 | 0 | 0 |
| Stomatitis | 27 | 5 | 5 | 0 |
| Paraesthesia | 22 | 3 | 0 | 0 |
| Allergy | 11 | 11 | 3 | 0 |
| Headache | 5 | 3 | 0 | 0 |
| Constipation | 8 | 5 | 3 | 0 |
| Myalgia | 11 | 8 | 0 | 0 |
| Local pain | 0 | 11 | 0 | 0 |
| Unpleasant taste | 5 | 3 | 0 | 0 |
| Cardiac dysrhythmia | 0 | 0 | 3 | 3 |
| Peripheral oedema/weight gain | 8 | 13 | 5 | 0 |
| Fever | 3 | 13 | 0 | 0 |

[a]Worst grade per patient.

**Table IV** Antitumour activity in 37 eligible patients

| | No. | % | Duration of response (months) Median | (range) |
|---|---|---|---|---|
| Partial remission | 8 | 22 | 7.5 | (3–11+) |
| No change | 11 | 30 | 4 | (3–8) |
| Progression | 14 | 38 | – | |
| Non-evaluable | 4 | 10 | – | |
| Total | 37 | 100 | | |

Table V Responders

| No. | Age | Sex | PS | No. of Taxotere cycles to PR | Indicator lesion | Percent decrease in tumour measurement | Duration of PR (months) | Survival (months) |
|---|---|---|---|---|---|---|---|---|
| 1 | 66 | M | 0 | 2 | Retroperitoneal lymph nodes | 61 | 4 | 6 |
| 2 | 38 | M | 2 | 2 | Mediastinal, presternal and supraclavicular masses | 94 | 8 | 10 |
| 3 | 44 | M | 2 | 4 | Retroperitoneal lymph nodes | 55 | 8 | 17 |
| 4 | 72 | F | 2 | 2 | Primary tumour | 84 | 3 | 3 |
| 5 | 68 | M | 2 | 2 | Skin nodules | 96 | 5 | 7 |
| 6 | 67 | M | 0 | 2 | Liver | 100[a] | 11+ | 11+ |
| 7 | 61 | M | 1 | 2 | Abdominal wall mass | 100[a] | 7+ | 7+ |
| 8 | 71 | M | 0 | 4 | Retroperitoneal lymph nodes | 90 | 10 | 11 |

[a]Non-measurable disease, not completely disappeared.

Weight gain of >2 kg was recorded in 8 of the 37 patients, all of whom had received four cycles or more of docetaxel. This was due to fluid retention, which resulted in peripheral oedema and sometimes pleural effusions (transudates). The median weight gain in these eight patients was 6.2 kg (range 2–13 kg).

Three cardiac events (two supraventricular arrhythmias in the form of paroxysmal atrial tachycardia and atrial fibrillation and one episode of coronary ischaemia) occurred in two patients while on docetaxel chemotherapy. The supraventricular arrhythmias occurred on day 4 of the first cycle and on day 8 of the second cycle of docetaxel, and subsided in both patients with conventional therapy. Both patients were uneventfully rechallenged with docetaxel.

### *Anti-tumour activity*

Eight patients achieved a partial remission; seven of these eight responses have been externally and independently reviewed and confirmed. The median duration of response was 7.5 months (range, 3 to 11 + months) (Table IV). Responses occurred in a variety of metastatic sites (Table V) including the liver (one patient), retroperitoneum (three patients), soft-tissue masses in the abdominal wall (one patient) and in the neck and mediastinum (one patient). One patient had multiple cutaneous nodules, and in another patient the primary tumour site responded to docetaxel. None of the remaining patients who had not undergone gastrectomy responded at the primary site.

Eleven patients had stabilisation of disease for a median of 4 months (range 3–8 months). Disease progression occurred in 14 patients, including three patients in whom progression was documented during the first 6 weeks of docetaxel chemotherapy ('early progression'). Response could not be assessed in the remaining four patients: one refused further docetaxel chemotherapy following the first administration, one patient died suddenly at home of unknown causes on day 14 of the first cycle, one proved to have no measurable lesion upon review and the fourth patient refused further treatment and evaluation after two courses.

The partial remission rate calculated for all 42 patients registered into the trial is 19%; it is 22% for the 37 eligible patients and 24% for the 33 evaluable patients.

Responses to docetaxel did not correlate with initial PS, age, metastatic sites, degree of myelosupression or the presence of an HSR to docetaxel. Seven of the eight responders were male. Six of 11 female patients, all PS 0–1, had stabilisation of disease while on docetaxel. Responses occurred more frequently in patients who underwent resection of the primary tumour than in those who did not: 7/19 (37%) *vs* 1/18 (5%) ($P = 0.06$).

### Discussion

The results of this clinical trial indicate that docetaxel is an active drug against gastric cancer. In fact, the response rate achieved with docetaxel is similar to the single-agent activity observed with the most active conventional drugs in use in this disease, such as 5-fluorouracil, doxorubicin, cisplatin and mitomycin C. Responses were usually observed after two courses of docetaxel and occurred in a variety of metastatic sites, including the liver, with a median duration of 7.5 months. Response at the primary tumour site was observed in only one patient. Patients who had undergone a gastrectomy with resection of the primary tumour had a higher response rate than patients whose tumour was not removed. Patient in this last category probably had more advanced disease with a larger tumour burden.

While most cycles of docetaxel were given according to the originally planned dose and schedule, a wide variety of side-effects were recorded. Neutropenia represented the major toxicity for most patients; nevertheless, there were only eight episodes (5%) of leucopenic fever requiring hospital admission, reflecting the prompt recovery of the leucocyte count.

The non-haematological side-effects included dermatological reactions as well as mild to moderate HSRs, which were subsequently controlled with antiallergic premedication and never led to the discontinuation of docetaxel. Some patients complained of fatigue and myalgias while on treatment with docetaxel. Most troublesome, however, was the development of a fluid retention syndrome, observed mainly in patients receiving four or more cycles of docetaxel and characterised by weight gain, peripheral oedema and pleural effusion. This syndrome has also been observed in other phase II trials with docetaxel carried out by the ECTG, a detailed description of which is reported elsewhere (Wanders *et al.*, 1993); preliminary data suggest that corticosteroids might benefit these patients to some extent.

There has been a growing disappointment with the use of drug combinations such as FAM (Biran *et al.*, 1989) or, more recently, EAP (Taal *et al.*, 1990) in the treatment of advanced gastric cancer, making the need for novel, active and, hopefully, less toxic combinations urgent. Recently, the use of paclitaxel in 20 patients with advanced adenocarcinomas of the upper gastrointestinal tract resulted in only one partial remission (Einzig *et al.*, 1993). In contrast, the present study does inidcate definite activity for docetaxel, and the difference may partly be explained by the extent of prior chemotherapy.

Further clinical investigations with docetaxel in patients with carcinoma of the stomach are certainly warranted based on the experience reported here, including the use of docetaxel in combination with other active drugs in this disease, such as the anthracyclines, 5-fluorouracil and others. Other possible venues for investigation include the routine use of premedication to circumvent HSRs and the fluid retention syndrome, and the concomitant administration of granulocyte colony-stimulating factor (G-CSF).

These investigations shall define the precise role of docetaxel in the overall treatment of gastric cancer.

### References

BIRAN, H., SULKES, A. & BIRAN, S. (1989). 5-Fluorouracil, doxorubicin (adriamycin) and mitomycin C (FAM) in advanced gastric cancer: observations on response, patient characteristics, myelosuppression and delivered dosage. *Oncology*, **46**, 83–87.

BISSERY, M.C., GUENARD, D. & GUERITTE-VOEGELEIN, F. (1991). Experimental antitumor activity of taxotere (RP 56976; NSC 628503), a taxol analog. *Cancer Res.*, **51**, 4845–4852.

BISSETT, D., CASSIDY, J. & SETANOIANS, A. (1992). Phase I study of taxotere administered as a 24-hour infusion. *Proc. Am. Assoc. Cancer Res.*, **33**, 526.

BRUNO, R., VERGNIOL, J.C. & MONTAY, G. (1992). Clinical pharmacology of taxotere (RP 56976) given as 1–2-hour infusion every 2–3 weeks. *Proc. Am. Assoc. Cancer Res.*, **33**, 261.

BURRIS, H., IRVIN, R., KUHN, J., KALTER, S., SMITH, L., SHAFFER, D., FIELDS, S., WEISS, G., ECKARDT, J., RODRIGUEZ, G., RINALDI, D., WALL, J., COOK, G., SMITH, S., VREELAND, F., BAYSSAS, M., LEBAIL, N. & VON HOFF, D. (1993). Phase I clinical trial of taxotere administered as either a 2-hour or 6-hour intravenous infusion. *J. Clin. Oncol.*, **11**, 950–958.

DE VALERIOLA, D., BRASSINNE, C. & PICCART, M. (1992). Phase I pharmacokinetic study of taxotere (RP 56976, NSC 628503) administered as a weekly infusion. *Proc. Am. Assoc. Cancer Res.*, **33**, 261.

EINZIG, A., WIERNIK, P.H., LIPSITZ, S. & BENSON III, A.B. (1993). Phase II trial of taxol in patients with adenocarcinoma of the upper gastrointestinal tract. The Eastern Cooperative Oncology Group Results. *Proc. Am. Soc. Clin. Oncol.*, **12**, 194.

GUERITTE-VOEGELEIN, F., GUENARD, D., LAVELLE, F., LEGOFF, M.T., MANGATAL, L. & POTIER, P. (1991). Relationships between the structure of taxol analogues and their antimitotic activity. *J. Med. Chem.*, **34**, 992–998.

KELSEN, D. (1988). Chemotherapy of gastric cancer: a review. *Isr. J. Med. Sci.*, **24**, 557–561.

MACDONALD, J.S., SCHEIN, P.S., WOOLLEY, P.V., SMYTHE, T., UENO, W., HOTH, D., SMITH, F., BOIRON, M., GISSELBRECHT, G., BRUNET, R. & LAGARDE, C. (1980). 5-Fluorouracil, mitomycin C and adriamycin (FAM): a new combination chemotherapy program for advanced gastric carcinoma. *Ann. Intern. Med.*, **93**, 533–540.

PAZDUR, R., NEWMAN, R.A. & NEWMAN, B.M. (1992). Phase I trial of taxotere (RP 56976). *Proc. Am. Assoc. Cancer Res.*, **11**, 111.

RINGEL, I. & HORWITZ, S.B. (1991). Studies with RP 56976 (taxotere): a semisynthetic analog of taxol. *J. Natl Cancer Inst.*, **83**, 2289–2291.

ROUSSEAU, F., EXTRA, J.M., CULINE, S., LOGE, N., LEBAIL, N. & MARTY, M. (1991). Phase I and pharmacologic study of taxotere, a new taxol analog. *Eur. J. Cancer*, **27** (Suppl. 2), 1238.

SILVERBERG, E. (1985). Cancer statistics CA-A. *Cancer J. Clin.*, **35**, 19–35.

TAAL, B.G., TEN BOKKEL HUININK, W.W., FRANKLIN, H. & RODENHUIS, S. (1990). EAP in advanced gastric cancer. *J. Clin. Oncol.*, **8**, 939–940.

TOMIAK, E., PICCART, M.J., KERGER, J., DEVALERIOLA, D., TUENI, E., LOSSIGNOL, D., LIPS, S., LEBAIL, N. & BAYSSAS, M. (1991). A phase I study of taxotere (RP 56976, NSC 628503) administered as a one hour intravenous (IV) infusion on a weekly basis. *Eur. J. Cancer*, **27** (Suppl. 2), 1184.

WANDERS, J., VAN OOSTEROM, A., GORE, M., PICCART, M., WOLFF, I., KAPLAN, S., ROELVINK, M., FRANKLIN, H., KAYE, S.B. & BAYSSAS, M. (1993). Taxotere toxicity – protective effects of premedication. *Eur. J. Cancer*, **29A** (Suppl. 6), 206.

WILS, J., BLEIBERG, H., DALESIO, O., BLIJHAM, G., MULDER, N., PLANTING, A., SPLINTER, T. & DUEZ, N. (1986). An EORTC Gastrointestinal group evaluation of the combination of sequential methotrexate and 5-fluorouracil combined with adriamycin in advanced measurable gastric cancer. *J. Clin. Oncol.*, **4**, 1799–1803.